# Concomitant experimental coinfection by *Plasmodium berghei* NK65-NY and *Ascaris suum* downregulates the *Ascaris*-specific immune response and potentiates *Ascaris*-associated lung pathology

**DOI:** 10.1186/s12936-021-03824-w

**Published:** 2021-07-01

**Authors:** Flaviane Vieira-Santos, Thaís Leal-Silva, Luiza de Lima Silva Padrão, Ana Cristina Loiola Ruas, Denise Silva Nogueira, Lucas Kraemer, Fabrício Marcus Silva Oliveira, Marcelo Vidigal Caliari, Remo Castro Russo, Ricardo Toshio Fujiwara, Lilian Lacerda Bueno

**Affiliations:** 1 Laboratory of Immunology and Genomics of Parasites, Institute of Biological Sciences, Department of Parasitology, Universidade Federal de Minas Gerais, Belo Horizonte, Brazil; 2 Laboratory of Protozooses, Institute of Biological Sciences, Department of General Pathology, Universidade Federal de Minas Gerais, Belo Horizonte, Brazil; 3 Laboratory of Pulmonary Immunology and Mechanics, Institute of Biological Sciences, Department of Physiology and Biophysics, Universidade Federal de Minas Gerais, Belo Horizonte, Brazil

**Keywords:** *Plasmodium berghei* NK65-NY, *Ascaris suum*, Coinfection, Malaria, Helminth infection, Lung inflammation, Lung injury, Pulmonary mechanics

## Abstract

**Background:**

Ascariasis and malaria are highly prevalent parasitic diseases in tropical regions and often have overlapping endemic areas, contributing to high morbidity and mortality rates in areas with poor sanitary conditions. Several studies have previously aimed to correlate the effects of *Ascaris-Plasmodium* coinfections but have obtained contradictory and inconclusive results. Therefore, the present study aimed to investigate parasitological and immunopathological aspects of the lung during murine experimental concomitant coinfection by *Plasmodium berghei* and *Ascaris suum* during larvae ascariasis.

**Methods:**

C57BL/6J mice were inoculated with 1 × 10^4^ *P. berghei* strain NK65-NY-infected red blood cells (iRBCs) intraperitoneally and/or 2500 embryonated eggs of *A. suum* by oral gavage. *P. berghei* parasitaemia, morbidity and the survival rate were assessed. On the seventh day postinfection (dpi), *A. suum* lung burden analysis; bronchoalveolar lavage (BAL); histopathology; NAG, MPO and EPO activity measurements; haematological analysis; and respiratory mechanics analysis were performed. The concentrations of interleukin (IL)-1β, IL-12/IL-23p40, IL-6, IL-4, IL-33, IL-13, IL-5, IL-10, IL-17A, IFN-γ, TNF and TGF-β were assayed by sandwich ELISA.

**Results:**

Animals coinfected with *P. berghei* and *A. suum* show decreased production of type 1, 2, and 17 and regulatory cytokines; low leukocyte recruitment in the tissue; increased cellularity in the circulation; and low levels of NAG, MPO and EPO activity that lead to an increase in larvae migration, as shown by the decrease in larvae recovered in the lung parenchyma and increase in larvae recovered in the airway. This situation leads to severe airway haemorrhage and, consequently, an impairment respiratory function that leads to high morbidity and early mortality.

**Conclusions:**

This study demonstrates that the *Ascaris-Plasmodium* interaction is harmful to the host and suggests that this coinfection may potentiate *Ascaris*-associated pathology by dampening the *Ascaris*-specific immune response, resulting in the early death of affected animals.

**Supplementary Information:**

The online version contains supplementary material available at 10.1186/s12936-021-03824-w.

## Background

Parasitic coinfections are common in different regions worldwide. Helminth infections, such as ascariasis, are present in all tropical regions, which are also areas with malaria transmission [1, 2]. The overlapping distribution of these parasites results in the occurrence of *Plasmodium* spp. and *Ascaris* spp. coinfections [3–6] that affect the outcome of these individual infections. Among the geohelminths that affect humans, *Ascaris* spp. has one of the highest infection rates by far, affecting approximately 800 million people worldwide [7–10]; they have a significant negative impact on human health and socioeconomic growth in affected populations [10].

Helminth parasites are notable for their capacity to modulate the parasite-directed host immune response [11, 12]; with chronic infection, the parasites modulate the host response to bystander antigens/pathogens [13–15] and allergic diseases [16, 17] and may suppress the actions of vaccines [18, 19]. In human ascariasis, as in most gastrointestinal nematode infections, the immune response is traditionally characterized by a highly polarized type 2 cytokine response in addition to high circulating levels of IgG1, IgG4 and total and specific IgE antibodies [20–22]. This type of Th2 response profile is associated with significant peripheral and tissue eosinophilia, accompanied by intense tissue mastocytosis [23]. However, the establishment of the chronic phase of infection has been associated with the development of type 1 responses at the same time as type 2 responses [24–26], which is considered crucial for the immune control of numerous viral, bacterial, or protozoal infections, such as *Plasmodium* spp., for which protection from infection is mediated by cytokines, IFN-γ and TNF [27, 28].

Malaria is a global disease with large endemic areas in sub-Saharan Africa, Asia and South America. *Plasmodium* spp. infections affect approximately 200 million people, resulting in more than 400,000 deaths each year [29]. *Plasmodium vivax*, *Plasmodium falciparum* and *Plasmodium knowlesi*-infected patients may develop malaria-associated acute respiratory distress syndrome (MA-ARDS), characterized by pulmonary oedema and haemorrhage [30–32]. In contrast with other complications of malaria, MA-ARDS pathology has a poor prognosis and remains poorly understood [32, 33].

Given the importance of these parasitic diseases in the context of public health, several epidemiological studies have aimed to understand how *Ascaris* spp. influences *Plasmodium* spp. infections [34]. However, recent findings are controversial, making it impossible to conclude whether the outcome of this interaction is beneficial, neutral or harmful to the host [4, 35].

In this study, a concomitant coinfection model of larvae ascariasis by *A. suum* [26, 36] and MA-ARDS by *P. berghei* [31, 37, 38] was used to characterize immunological, pathological and physiological aspects of pulmonary pathology. This work study demonstrates that the *Ascaris-Plasmodium* interaction is harmful to the host, resulting in low responsiveness of the animals to increased injury and loss of lung function due to increased migration of larvae into the lung, causing the early death of the animals.

## Methods

### Experimental design

Seven-week-old male C57BL/6J mice, considered susceptible to *P. berghei* (strain NK65-NY) infection [37, 39], and *Ascaris* spp. [26, 40] were obtained from the Biotério Central of the Federal University of Minas Gerais and used in the study. During the experimental period, the mice were provided filtered water and commercial chow (Nuvilab Cr-1, Nuvital Nutrients, Brazil) ad libitum. They were housed in cages (50 × 60 × 22 cm) with sterile sawdust shavings, which were changed and cleaned once a week. Mice were maintained in the Animal Facility of the Laboratory of Immunology and Genomics of Parasites of the Federal University of Minas Gerais under controlled conditions of temperature (24 ± 1 °C) and lighting (12-h light–dark cycle).

**Fig. 1:**
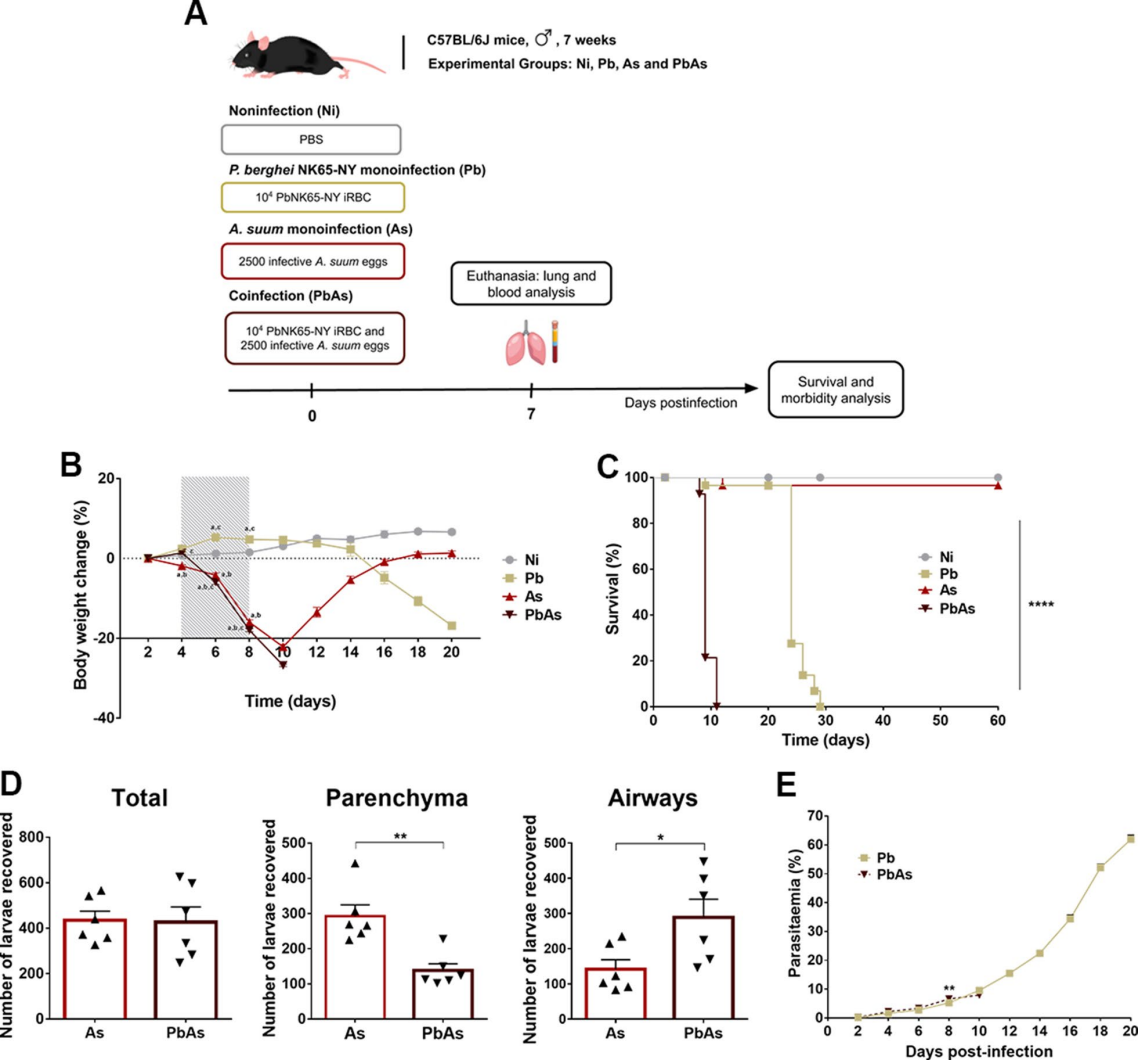
Clinical and parasitological aspects of *P. berghei* and *A. suum* coinfection. **A** Experimental design. **B** Variation of body weight was expressed as a percentage from the initial weight of mice. The hatched area represents the times when statistical analysis was carried out between all groups. **C** Survival rate. **D** Larvae parasitic load in the pulmonary parenchyma and BAL on 7th day postinfection (dpi) (n = 6 per group). **E** Parasitaemia levels. Circles—noninfected group (n = 6), Square—*P. berghei* NK65-NY mono-infected group (n = 15), Triangle—*A. suum* mono-infected group (n = 15), Inverted triangle—coinfected group (n = 15). Survival curves were compared using the Gehan–Breslow–Wilcoxon test. Larvae burdens were compared using the Mann–Whitney test. Two-way ANOVA test followed by multiple comparisons test was used to evaluate variations in body weight and parasitaemia. The results are shown as the mean ± SEM and significant differences (p ≤ 0.05) among the groups are represented by (*a*) NI group, (*b*) Pb group and (*c*) As group or *p < 0.05, **p < 0.01 and ****p < 0.0001

The mice were divided into four groups: noninfected animals (Ni); *A. suum-*monoinfected animals (As); *P. berghei*-monoinfected animals (Pb); and *P. berghei* and *A. suum*-coinfected animals (PbAs). Mono- and coinfections were performed simultaneously on the first day of the experiment (t = 0) (Fig. 1A). Six or eight mice/group of control and infected mice, respectively, were euthanized for each experiment with a lethal dose of anesthetic (ketamine 390 mg/kg and xylazine 27 mg/kg) on the seventh day after infection, which is considered to be the period when *Ascaris* larvae migration peaks in the lungs [26, 36].

### Ethics statement

The maintenance and use of mice were carried out in accordance with the recommendations of the Brazilian College of Animal Experimentation (COBEA). The present study was submitted to and approved by the Ethics Committee for Animal Experimentation (CEUA) of the Federal University of Minas Gerais, Brazil, under protocol n. 21/2018.

### Parasites and experimental infections

*Plasmodium berghei* NK65-NY, which is a well-known MA-ARDS model [31, 37, 38], was kindly provided by Dr. Antoniana Ursine Krettli of René Rachou-FIOCRUZ/MG Malaria Laboratory and was maintained in BALB/c mice in weekly blood passages at the Animal Facility of the Laboratory of Immunology and Genomics of Parasites–UFMG/MG.

*Ascaris suum* adult worms were collected from the intestines of infected pigs that were discarded by a slaughterhouse located in the city of Belo Horizonte, Minas Gerais, Brazil. Adult worms were kept in PBS (0.4 M NaCl and 10 mM NaPO_4_) and taken to the Laboratory of Immunology and Genomics of Parasites of the Federal University of Minas Gerais to be processed. The eggs were isolated from the uteruses of female adult worms by mechanical maceration, purified by filtration with 100-μm nylon strainers, placed in culture bottles with 50 mL of 0.2 M H_2_SO_4_ at a concentration of 25 eggs/μL and maintained in a BOD incubator at 26 °C. On the 150th day of culture, at the peak of larvae infectivity, fully embryonated eggs were used for experimental infections [26].

Mono- and coinfected animals were inoculated intraperitoneally with phosphate-buffered saline (PBS) containing 1 × 10^4^ *P. berghei* iRBCs and/or 200 μL of PBS with 2500 embryonated eggs of *A. suum* by oral gavage, as previously described [26, 36]. Noninfected animals were inoculated only with 100 μL of PBS.

### Survival curve

To evaluate the course of coinfection, the survival of animals belonging to the experimental groups was evaluated every two days (n = 15 for each group of infected animals and n = 6 for noninfected animals). All animals were followed for up to 60 days, during which they were evaluated for mortality, weight variation and *P. berghei* parasitaemia.

### Parasitological analysis

Parasitaemia was monitored during the survival curve analysis and determined by staining blood smears from mice tails with Giemsa and observation with light microscopy. Slides were coded, and iRBC percentages were calculated by counting the number of infected red blood cells in a total of 1000 red blood cells.

The parasite burden of *Ascaris* was evaluated by the recovery of larvae from the lungs and BAL on the seventh day postinfection (dpi), since this is the larvae peak of *Ascaris* in the lung [26]. Tissues were collected, cut with scissors and placed in a modified Baermann apparatus for 4 h in PBS at 37 °C. The recovered larvae were fixed (with 10% formaldehyde in PBS) and counted under an optical microscope [26, 36, 41].

### Haematological analysis

The animals were anesthetized (ketamine 100 mg/kg/xylazine 09 mg/kg) to collect 500 μL of blood from the retro-orbital plexus using a Pasteur capillary pipette. The collected blood was transferred to tubes containing EDTA anticoagulant. Subsequently, the tubes were centrifuged to collect plasma and were stored at − 80 °C until further analysis. Total counts of erythrocytes, leukocytes and platelets and haemoglobin levels were determined using an automated haematological analyzer (Bio-2900 Vet, Bioeasy, USA). For differential white blood cell counts, blood smears were stained with Giemsa, and 100 white blood cells were counted under a light microscope.

### Bronchoalveolar lavage analysis

BAL was performed by inserting a 1.7-mm catheter into the trachea of mice, and one milliliter of PBS was flushed twice through the catheter to collect BAL. The material was centrifuged at 300×*g* for 10 min at 4 °C, and the pellet was used to determine total and differential cellularity using optical microscopy. The supernatant was used to quantify the amount of total protein and haemoglobin content. Samples from noninfected mice were used as controls.

The extent of alveolar haemorrhage was assessed based on the amount of haemoglobin (Hb) detected in BAL supernatant using the Drabkin method according to the manufacturer's instructions (Bioclin, Brazil). The concentration was determined spectrophotometrically by measuring the absorbance at 540 nm. Haemoglobin content is expressed as g/dL Hb per mL BAL. Total protein quantification was performed with a BCA Protein Assay Kit (Thermo Scientific, USA) and was performed on BAL to measure possible protein leakage into the airways, as previously described [36]. The results are expressed as μg of total protein per mL of BAL.

### Pulmonary cytokine profile

To examine the cytokine profile, the lungs from mice in all experimental groups were removed and homogenized (TissueLyser LT, Qiagen, Germany) in extraction solution (0.4 M NaCl, 0.05% Tween 20, 0.5% BSA, 0.1 mM phenylmethylsulfonyl fluoride, 0.1 mM benzethonium chloride, 10 mM EDTA and 20 KI units aprotinin) in a volume of 1 mL per 100 mg of lung tissue. The resulting homogenates were centrifuged at 1500×*g* for 10 min at 4 °C, and the supernatants were collected and stored at − 80 °C. The concentrations of IL-1β, IFN-γ, IL-12/IL-23p40, IL-6, TNF, IL-4, IL-33, IL-13, IL-5, IL-10, TGF-β and IL-17A were assayed by sandwich ELISA kits (R&D Systems, USA) according to the manufacturer's instructions. The absorbance was determined by a VersaMax ELISA microplate reader (Molecular Devices, USA) at a wavelength of 492 nm. The cytokine concentration (pg/mL) for each sample was calculated by interpolation from a standard curve. All samples were tested in duplicate.

### Macrophage *n*-acetylglucosaminidase, neutrophil myeloperoxidase and eosinophil peroxidase assays

The activities of macrophage *N*-acetylglucosaminidase (NAG), neutrophil myeloperoxidase (MPO) and eosinophil peroxidase (EPO) in pulmonary homogenates were detected according to a previously described method [36, 42]. After tissue homogenization, the homogenate was centrifuged at 1500*g* for 10 min at 4 °C, and the resulting pellet was examined to determine NAG, MPO and EPO activities. Absorbance was determined by a VersaMax ELISA Microplate Reader (Molecular Devices, USA) according to the protocol for each assay, and the results are expressed as the optical density (O.D.).

### Histopathological and morphometric analysis

The left lobe of the lung was removed from the mice in each group. The organs were fixed in 10% formalin solution, gradually dehydrated in ethanol before being diaphanized in xylol, and embedded in paraffin blocks that were cut at a thickness of 4 μm and fixed on microscopy slides. Slides with lung tissue were stained with haematoxylin and eosin, and the lesions in the pulmonary parenchyma were described in terms of the lesion intensity, inflammation, and vascular phenomena.

To examine lung inflammation based on peribronchial inflammation, perivascular inflammation, parenchymal inflammation and the haemorrhage score, ten random images were captured per animal and analysed (10× magnification). The score was created by adapting the methodology previously described by Horvat et al. [43] (see Additional file 1: Table S1).

To assess the pulmonary inflammation intensity, the degree of interalveolar septa thickening was calculated. Twenty random images were captured with a 20× magnification objective using a microscope camera (TK-1270/RGB, JVC, Japan), during which a lung area of 3.2 × 10^6^ mm^2^ was analysed. Tissues were examined using KS300 software coupled with a image analyzer (Zeiss, Germany), where all lung tissue pixels in the real image were selected for binary image creation, digital processing and area calculation in mm^2^ of interalveolar septum [36, 44].

### Assessment of respiratory mechanics

The evaluation of pulmonary function and physiology was performed by spirometry, as previously described [36, 41, 44]. Briefly, mice received an intraperitoneal injection of anesthesia (ketamine 100 mg/kg/xylazine 09 mg/kg) to maintain spontaneous breathing, were tracheostomized, placed in a plethysmograph and connected to a computer-controlled ventilator (Forced Pulmonary Maneuver System, Buxco Research Systems, USA). First, each anesthetized mouse was ventilated at a rate of 160 breaths per minute. After 3 min of ventilation, the constant-phase model was used to measure dynamic compliance (Cdyn) and lung resistance (RI). To measure the inspiratory capacity (IC), the quasi-static pressure–volume maneuver was performed, which inflates the lungs to a standard pressure of + 30 cm H_2_O and then slowly exhales until reaching a negative pressure of -30 cm H_2_O, thereby measuring the volume at each point of application in the lungs. A fast-flow volume maneuver was performed, and the lungs were first inflated to + 30 cm H_2_O and immediately afterwards were connected to a highly negative pressure to force expiration until − 30 cm H_2_O was reached. The forced vital capacity (FVC), forced expiratory volume (forced expiratory volume at 100 ms, FEV100) and Tiffeneau index (FEV 50/FVC) were recorded. Suboptimal maneuvers were discarded, and for each test in every single mouse, at least three acceptable maneuvers were conducted to obtain a reliable mean for all numeric parameters. After this experimental procedure, the animals were euthanized by exsanguination.

### Statistical analysis

GraphPad Prism 7 (GraphPad software, Inc., USA) was used for statistical analysis. Grubb’s test was used to detect sample outliers in all the results. To verify the distribution of data, the Shapiro–Wilk normality test was used. To compare larvae burdens, the Mann–Whitney test was used. To compare variations in body weight and the parasitaemia of *P. berghei*, two-way ANOVA followed by Tukey’s and Holm-Sidak’s multiple comparison tests were used. Data from histopathological semiquantitative analysis and BAL cellularity were analysed by the Kruskal–Wallis test followed by Dunn’s test. Data from haematological profiles; the morphometric analysis of septum thickness; NAG, MPO and EPO assays; protein and haemoglobin levels of BAL fluid; cytokine profiles; and pulmonary mechanics were analysed using one-way ANOVA followed by Tukey’s multiple comparison test. Survival curves were compared using the Gehan–Breslow–Wilcoxon test. All tests were considered significant at p ≤ 0.05.

## Results

### Coinfection by *P. berghei* and *A. suum* leads to increased morbidity and mortality and accelerates larvae migration to the lungs

To assess the impact of *Plasmodium-Ascaris* coinfection on the host, C57BL/6 J mice were simultaneously infected with 10^4^ iRBCs containing *P. berghei* or 2500 fully embryonated *A. suum* eggs (Fig. 1A). Initially, the morbidities of the noninfected, monoinfected and *Plasmodium-Ascaris*-coinfected groups were measured by the body weight loss index during 20 days of infection. The results showed higher morbidity in the coinfected mice, which was characterized by earlier weight loss, than in the *P. berghei*-monoinfected group (Fig. 1B). Coinfected animals began to lose weight at approximately 6 dpi, which persisted until their spontaneous death at 10 dpi. *A. suum*-monoinfected animals also had a large loss of body weight, similar to animals in the coinfected group; however, the weight of these monoinfected animals recovered at approximately 10 dpi. On the other hand, *P. berghei*-monoinfected mice began to lose weight a few days later (24 dpi) than mice in the coinfected group. Control animals presented a continuous increase in body mass throughout the observation period.

In addition, coinfected animals also died earlier than animals in the other groups. Coinfected animals started to die at 8 dpi, which continued until 11 dpi, with a peak at 9 dpi of more than 65% lethality (Fig. 1C). Monoinfected-*A. suum* mice did not present significant lethality. *Plasmodium berghei* monoinfection also lead to high lethality rates; however, the lethality occurred later than in the coinfected group.

To determine whether concomitant coinfection influenced the *Ascaris* parasitic burden, lung larvae were recovered on 7 dpi. The results showed that although there was no difference in the total larvae recovered from the lungs, coinfection lead to a significant decrease in larvae recovered from the lung parenchyma and a significant increase in larvae recovered from the airway compared to that in the *A. suum*-monoinfected group (Fig. 1D). Regarding the evolution of *P. berghei* parasitaemia, no differences were observed. These results revealed that concomitant coinfection did not alter the progression of *Plasmodium* parasitaemia compared to that in the monoinfected group (Fig. 1E). In summary, these results showed that coinfection induces high morbidity and early mortality. In addition, coinfected mice showed an increase in *Ascaris* larvae migration from the lung parenchyma to the airways, suggesting that more larvae would be able to complete the cycle. This change may be associated with the presence of *Plasmodium*, which might have altered the immune response and prevented the larvae from being contained.

### Haematological profile of mice with *P. berghei* and *A. suum* coinfection

Haematological analysis showed significant increases in total circulating leukocytes, lymphocytes, monocytes, and neutrophils in the coinfected group relative to the other groups (Table 1). Eosinophil counts were significantly increased in *A. suum*-monoinfected mice compared to *P. berghei*-monoinfected and noninfected mice. The analysis of red blood cell compartments showed significant reductions in total erythrocyte counts and haemoglobin levels in the coinfected group compared to the *A. suum*-monoinfected and noninfected groups. Regarding platelet counts, there was a significant decrease in coinfected animals compared to the monoinfected animals, suggesting that malaria-induced thrombocytopenia countered *Ascaris*-elevated platelet counts.

**Table 1:** Peripheral blood cells of *P. berghei* NK65-NY and/or *A. suum-*infected C57BL/6J mice at the 7 dpi

	Experimental groups			
	Ni Mean ± SD	Pb Mean ± SD	As Mean ± SD	PbAs Mean ± SD
Total leukocytes (× 10^3^/μL)	4.67 ± 1.43	10.92 ± 9.26	4.18 ± 1.45	18.75 ± 2.80^a,b,c^
Lymphocyte (× 10^3^/μL)	4,09 ± 1,27 (85–91%)	9.06 ± 7.53 (80–88%)	2.66 ± 1.14^b^ (52–77%)	14.39 ± 1.87^a,b,c^ (71–83%)
Monocyte (× 10^3^/μL)	0.20 ± 0.16 (2–6%)	1.06 ± 1.0 (1–11%)	0.26 ± 0.18 (2–10%)	1.11 ± 0.48^a,b,c^ (4–8%)
Neutrophil (× 10^3^/μL)	0.36 ± 0.17 (5–11%)	0.79 ± 0.17 (6–14%)	1.21 ± 0.50 (20–40%)	3.22 ± 1.42^a,b,c^ (11–23%)
Eosinophil (× 10^3^/μL)	0.01 ± 0.01 (0–1%)	0.00 ± 0.00 (0–1%)	0.06 ± 0.05^a,b^ (1–3%)	0.03 ± 0.01^c^ (0–1%)
Erythrocyte (× 10^6^/μL)	8.47 ± 0.60	7.47 ± 0.92	8.81 ± 2.74^a,b^	7.06 ± 0.65^a,c^
Haemoglobin (g/dL)	16.18 ± 1.10	14.17 ± 1.72	16.87 ± 5.05^a,b^	13.90 ± 1.15^a,c^
Platelet (× 10^3^/μL)	320 ± 133	111 ± 37^a^	538 ± 90^a,b^	274 ± 114^b,c^

One-way ANOVA followed by Tukey's multiple comparisons test were used to assess differences between groups. Results are presented as mean ± SD and significant differences (p ≤ 0.05) among the groups are represented by (a) NI group, (b) Pb group and (c) As group. (n = 6 for all groups)

### Coinfection by* P. berghei* and *A. suum* decreased leukocyte recruitment and cellular activity in the lungs but exacerbated haemorrhage in the airways, which might be associated with an increase in larvae migration

**Fig. 2:**
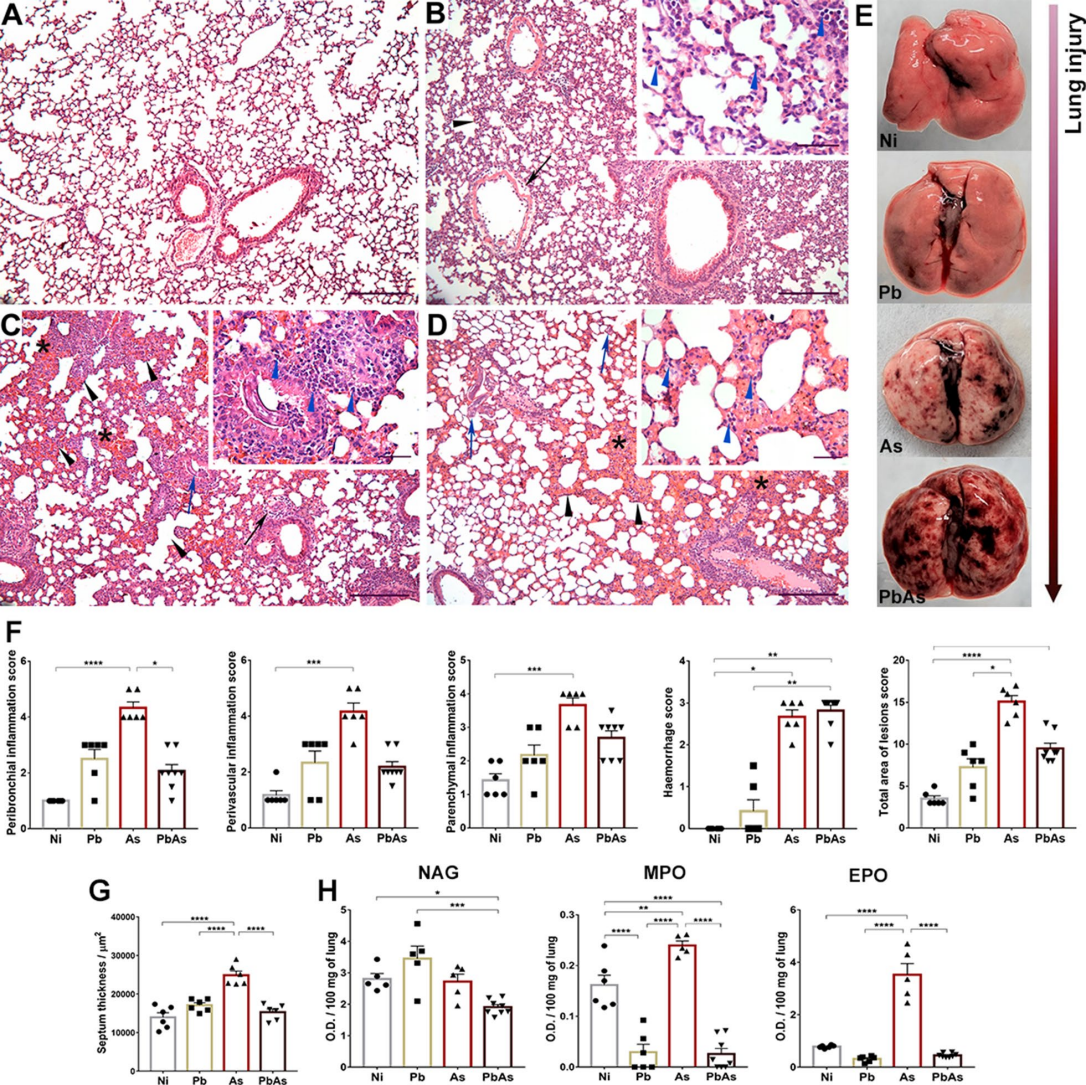
Lung injury and inflammation by *P. berghei* NK65-NY *and A. suum* coinfection at the 7 dpi. Histopathological analysis: **A** Panoramic image of the pulmonary parenchyma of the Ni group; **B** Panoramic image of the Pb group pulmonary parenchyma with slight thickening of the interalveolar septa (black arrowheads) and perivascular oedema (black arrow) with insert showing details: lymphocytes (blue arrowheads); **C** Panoramic image of group As pulmonary parenchyma with intense thickening of the interalveolar septa (black arrowheads), perivascular oedema (black arrow), *A. suum* larvae (blue arrow) and haemorrhage (*) with insert from the previous image showing details: eosinophils and neutrophils (blue arrowheads); **D** Panoramic image of the pulmonary parenchyma of the PbAs group with slight thickening of the interalveolar septa (black arrowheads) and haemorrhage (*) with insert from the previous image showing details: lymphocytes and macrophages (blue arrowheads). Haematoxylin & eosin stain. Scale bar of lowest magnification = 100 μm. Scale bar of highest magnification = 20 μm. **E** Macroscopy aspects of lungs of *P. berghei* and/or *A. suum-*infected mice. **F** Lung inflammation and haemorrhage score. **G** Septum thickness morphometry. **H** Cellular activity of macrophages (NAG), neutrophils (MPO), and eosinophils (EPO). Circles—non-infected group (n = 6), Square—*P. berghei* mono-infected group (n = 6), Triangle—*A. suum* mono-infected group (n = 6), Inverted triangle—coinfected group (n = 6 to 8). Kruskal–Wallis test followed by Dunn’s test and one-way ANOVA test followed by Tukey’s multiple comparisons were used. Results are shown as the mean ± SEM and were represented by *p < 0.05, **p < 0.01, ***p < 0.001; and ****p < 0.0001

Histopathological analysis of the pulmonary parenchyma allowed the observation and description of lesions caused by *P. berghei* and/or *A. suum* infections. *Plasmodium berghei-*monoinfected mice presented areas of lesions with perivascular oedema, haemorrhagic zones, and the hypertrophy and hyperplasia of epithelial cells of the bronchi and bronchioles (Fig. 2B) in addition to moderate and diffuse lymphocytic inflammatory infiltrate through the pulmonary parenchyma. During *Ascaris* infection, the presence of exudative phenomena, such as perivascular edema and haemorrhagic areas with the presence of scattered larvae in the pulmonary parenchyma, was frequently observed (Fig. 2C). Areas with mixed inflammatory infiltrate composed of eosinophils and neutrophils and, less frequently, of lymphocytes and macrophages were also frequently observed in this group. Coinfected mice presented exudative phenomena such as perivascular oedema, congested vessels and exuberant haemorrhagic areas, which were observed more frequently than in the *A. suum*-monoinfected group (Fig. 2D). A mild, mixed and diffuse inflammatory infiltrate in the pulmonary parenchyma was composed of lymphocytes, macrophages and, less commonly, neutrophils. In addition, all coinfected animals had many larvae in the pulmonary parenchyma. The hypertrophy and hyperplasia of epithelial cells of the bronchi and bronchioles were frequently observed in this group. All these injury phenomena were macroscopically observed in infected mice, where *A. suum*-monoinfected and coinfected mice presented reddish areas in the lungs, which were suggestive of lesions caused by larvae migration in the organs in these groups (Fig. 2E).

In accordance with these data, *A. suum*-monoinfected mice had increased inflammation compared to mice in the other groups. These animals also presented an increase in parenchymal pulmonary haemorrhage scores compared to mice in the control group and *P. berghei*-monoinfected group but similar to mice in the coinfected group (Fig. 2F). Consequently, *Ascaris*-monoinfected mice had increased interalveolar septum thickening compared to mice in the other groups (Fig. 2G). Corroborating previous observations [36], there were significant increases in MPO and EPO activities in *A. suum*-monoinfected mice compared to mice in the other groups (Fig. 2H). *Plasmodium berghei-*monoinfected animals had significant decreases in MPO and EPO activities compared *A. suum*-monoinfected and control animals. Regarding coinfection, significantly reduced NAG, MPO and EPO activities were observed in coinfected mice (Fig. 2H).

**Fig. 3:**
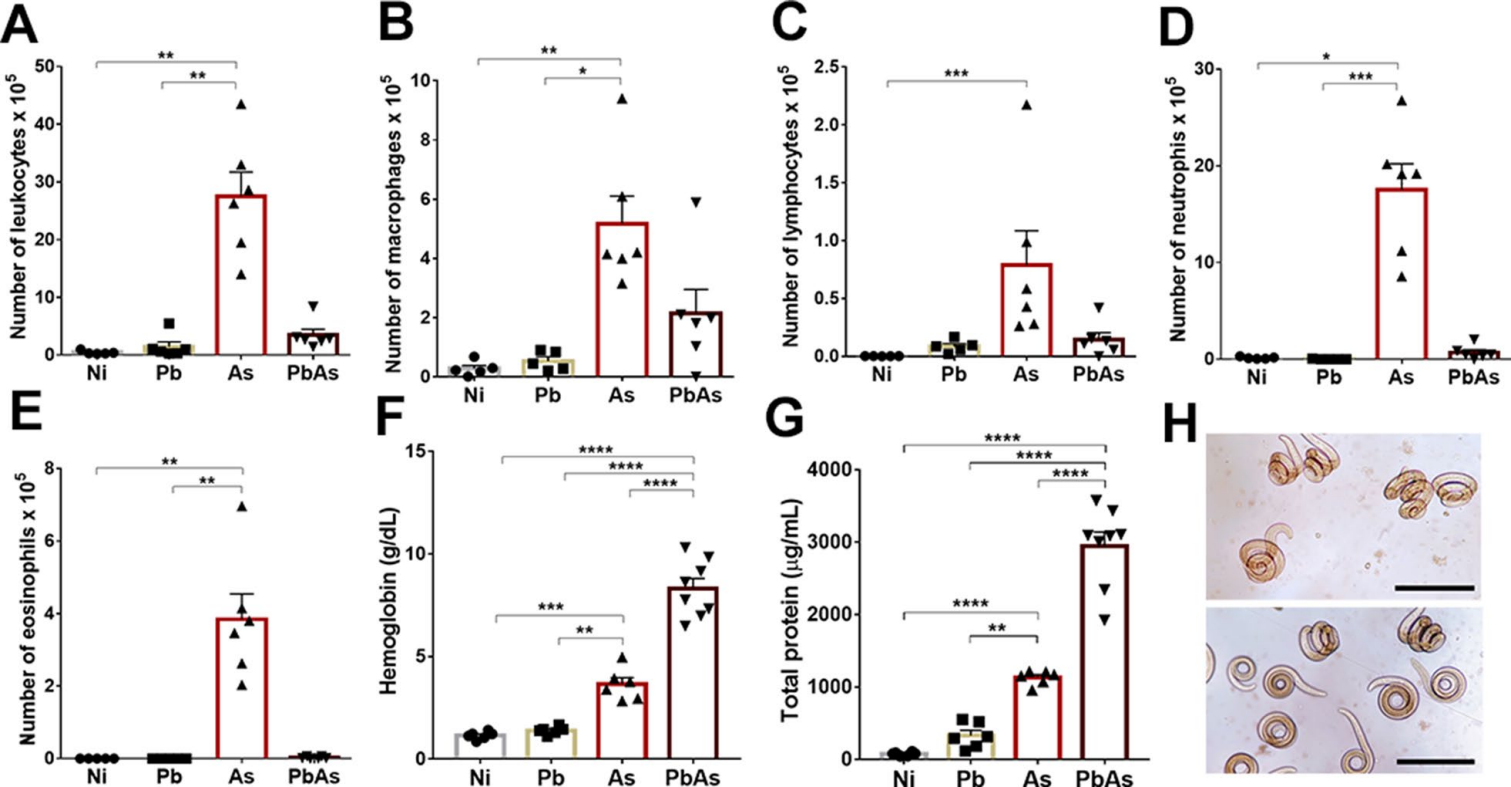
BAL analysis of *P. berghei* and *A. suum* coinfection at the 7 dpi. **A** Total leukocyte in BAL. **B** BAL macrophages. **C** BAL lymphocytes. **D** BAL neutrophils. **E** BAL eosinophils. **F** BAL haemoglobin levels. **G** BAL total protein levels. **H** Larvae recovered from the BAL of *Ascaris* mono-infected and coinfected mice. Scale bar = 100 μm. Circle—noninfected group (n = 6), Square—*P. berghei* NK65-NY mono-infected group (n = 6), Triangle—*A. suum* mono-infected group (n = 6), Inverted triangle—coinfected group (n = 6 to 8). Kruskal–Wallis test followed by Dunn’s test and one-way ANOVA test followed by Tukey’s multiple comparisons were used. Results are shown as the mean ± SEM and were represented by *p < 0.05, **p < 0.01, ***p < 0.001; and ****p < 0.0001

A similar profile was observed when analyzing the cellularity of the airways. Coinfected mice had a significant decrease in BAL cellularity (Fig. 3A–E). This was shown by the significant increase in the total number of leukocytes in *A. suum*-monoinfected mice compared to mice in the other groups (Fig. 3A). Regarding the cell subtypes, the numbers of macrophages, lymphocytes, neutrophils and eosinophils were also significantly increased in *A. suum*-monoinfected mice (Fig. 3B–E). In contrast, there were significant increases in haemoglobin (Fig. 3F) and total protein levels (Fig. 3G) in the BAL of coinfected animals compared to animals in the other groups, along with higher numbers of larvae in the airways compared to those in *A. suum*-monoinfected animals (Figs. 1D, 3H). Collectively, these results suggest that coinfection results in less leukocyte infiltration and activity in the lung parenchyma, a decrease in BAL cellularity and higher levels of haemoglobin in the airway, which might be associated with the rupture of blood vessels caused by the increased migration of larvae in the lungs of coinfected mice.

### Characterization of *P. berghei* and *A. suum* coinfection cytokine profiles in the lungs

**Fig. 4:**
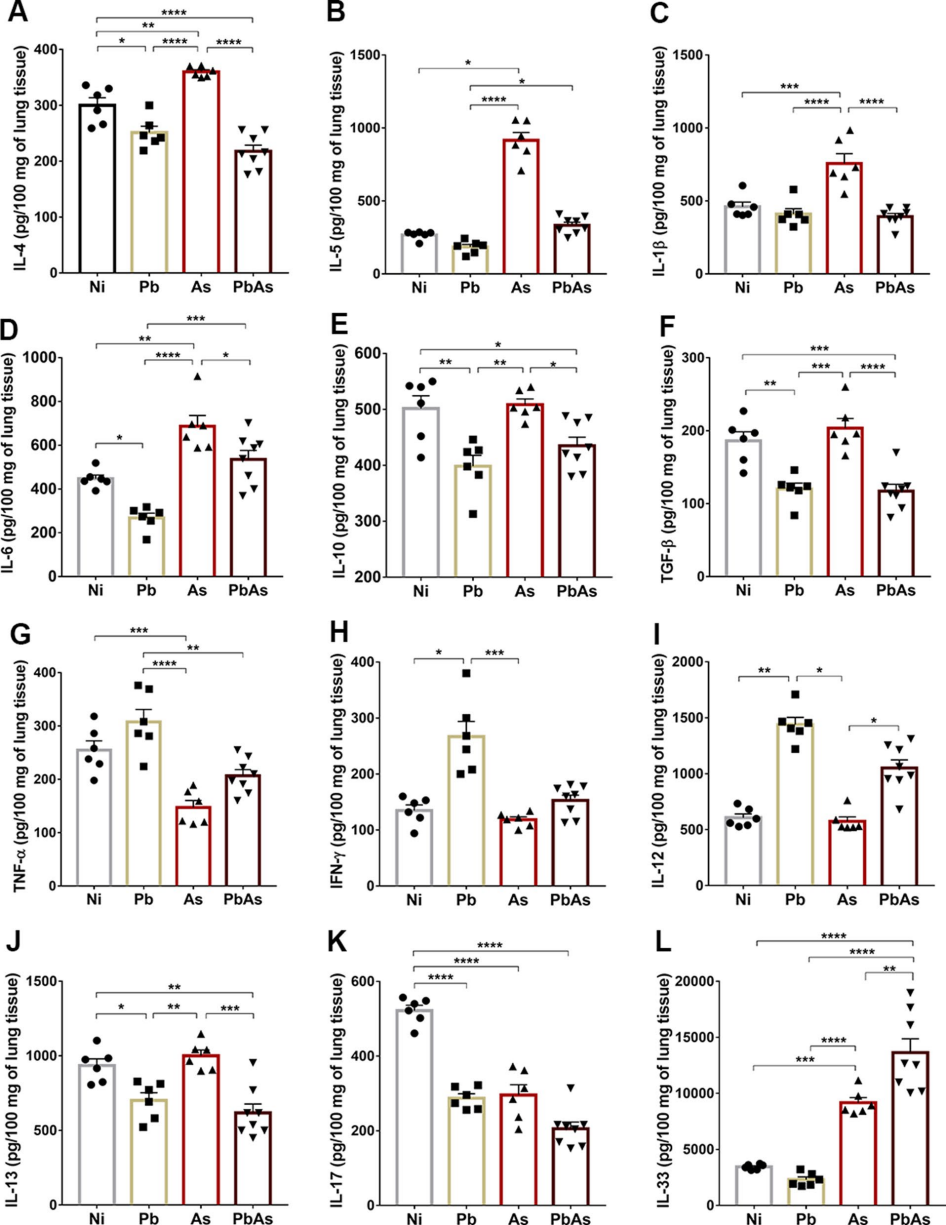
Pulmonary cytokine levels induced by *P. berghei and A. suum* coinfection at the 7 dpi. **A** IL-4; **B** IL-5; **C** IL-1β; **D** IL-6; **E** IL-10; **F** TGF- β; **G** TNF; **H** IFN- γ; **I** IL-12/IL-23p40; **J** IL-13; **K** IL-17-A; **L** IL-33. Circle—noninfected group (n = 6), Square—*P. berghei* NK65-NY mono-infected group (n = 6), Triangle—*A. suum* mono-infected group (n = 6), Inverted triangle—coinfected group (n = 8). One-Way ANOVA test followed by multiple comparisons test were used to compare the variances between the groups. The results are shown as the mean ± SEM and are represented by *p < 0.05, **p < 0.01, ***p < 0.001, and ****p < 0.0001

Host pulmonary cytokine production was characterized at 7 dpi. In *A. suum*-monoinfected animals, there were significantly higher levels of IL-4, IL-5, IL-1β and IL-6 cytokines than in animals in the other groups (Fig. 4A–D) and an increase in IL-10 and TGF-β regulatory cytokines compared to animals in the *Plasmodium*-monoinfected and coinfected groups (Fig. 4E, F). Additionally, *P. berghei-*monoinfected mice had significantly increased levels of TNF, IFN-γ, and IL-12 compared to *Ascaris*-monoinfected and noninfected animals, as expected (Fig. 4G–I) [28, 39]. However, the immune response of coinfected mice was characterized by significantly lower levels of IL-4, IL-5, IL-1β, IL-6, IL-10, TGF-β, IL-13 and IL-17 than that of the *Ascaris*-monoinfected mice (Fig. 4A–F, J, 4K). Interestingly, this profile was very similar to that of *Plasmodium*-monoinfected mice, except for IL-6 and IL-33 production (Fig. 4D and L), which were significantly higher in coinfected animals. The increased production of IL-6 and IL-33 might be explained by the influence of the host’s response to *Ascaris* infection. As seen in previous studies [26, 36, 45], the presence of IL-6 and IL-33 is important for restraining *Ascaris* larvae during migration. Thus, the increase in *Ascaris* larvae migration in the lungs of coinfected mice (Fig. 1D) may have influenced the increase in these cytokines in these animals. These data suggest that the overall immune response elicited during coinfection seems to be driven by *Plasmodium* infection.

### Coinfection by* P. berghei* and *A. suum* intensifies *Ascaris*-associated respiratory dysfunction

**Fig. 5:**
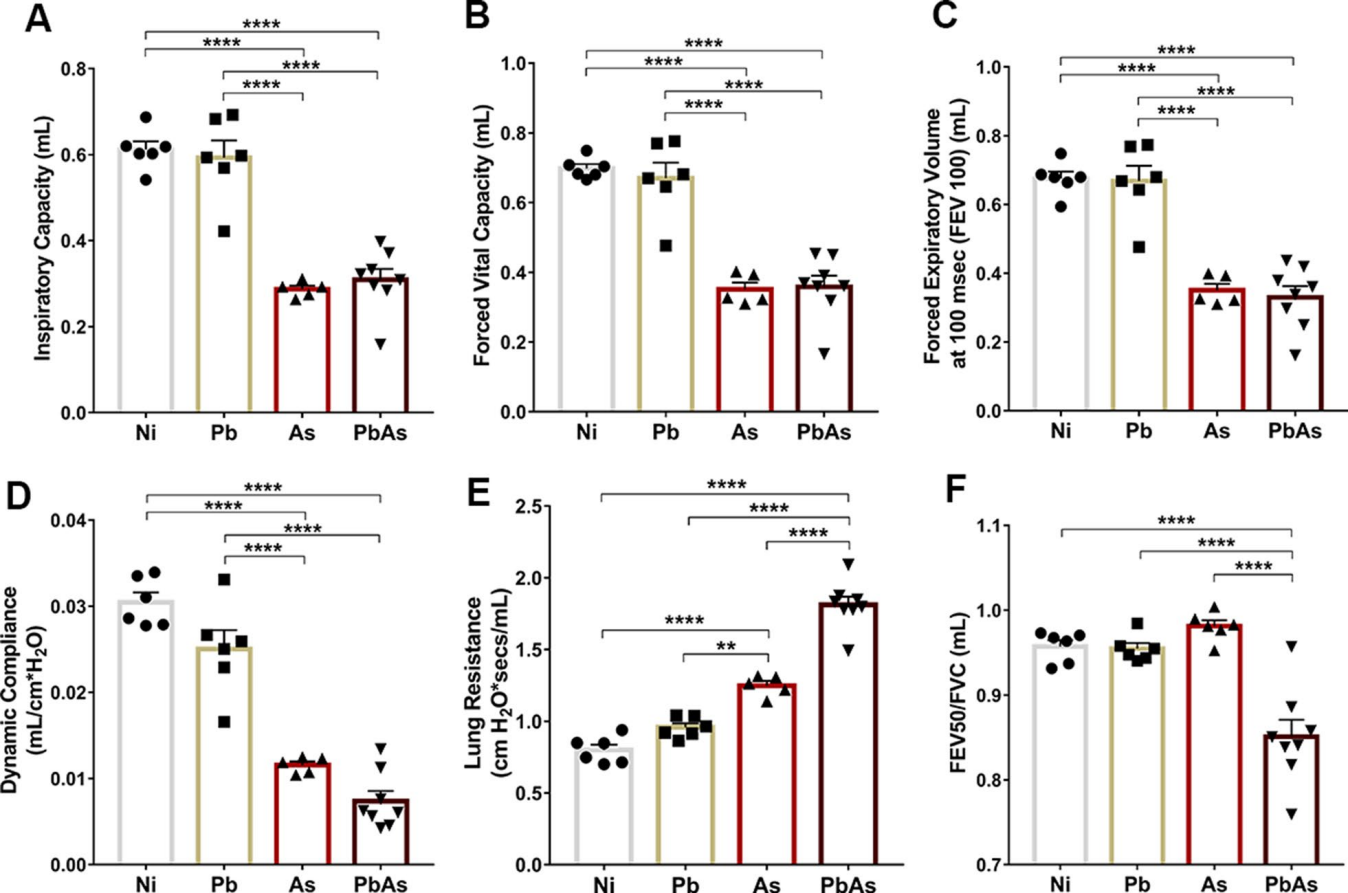
Increase in respiratory dysfunction in *P. berghei* NK65-NY *and A. suum* coinfected mice at the 7dpi. The parameters evaluated were: **A** Inspiratory capacity; **B** Forced vital capacity; **C** Forced expiratory volume; **D** Dynamic compliance; **E** Lung resistance; and **F** Tiffeneau index. Circle—noninfected group (n = 6), Square—*P. berghei* NK65-NY mono-infected group (n = 6), Triangle—*A. suum* mono-infected group (n = 6), Inverted triangle—coinfected group (n = 8). One-Way ANOVA test followed by Tukey’s multiple comparison test were used. The results are shown as the mean ± SEM and are represented by *p < 0.05, **p < 0.01, ***p < 0.001; and ****p < 0.0001

Pulmonary mechanics were evaluated by forced spirometry on a mechanical respirator to investigate physiological dysfunction. In this analysis, it was possible to verify the influence of larvae migration on the pulmonary physiology of coinfection. Coinfected mice exhibited significant reductions in inspiratory capacity (Fig. 5A) and forced vital capacity (Fig. 5B) and presented changes in respiratory flow with decreased forced expiratory volume (Fig. 5C) and lower dynamic compliance (Fig. 5D) compared to *P. berghei*-monoinfected and noninfected mice, but there were no significant differences relative to *A. suum*-monoinfected mice. Interestingly, two parameters were more pronounced in coinfected animals: higher pulmonary resistance (Fig. 5E) and air flow limitation, as shown by the decrease in the Tiffeneau index (Fig. 5F), than animals in the other groups and even compared to *A. suum*-monoinfected mice. Together, these results show that *Ascaris*-associated lung pathology is more enhanced in coinfected animals as a consequence of increased larvae migration in the lungs.

## Discussion

*Ascaris* spp. and *Plasmodium* spp. infections are prevalent in all tropical regions worldwide and are of great importance to public health. Numerous studies have already demonstrated the capacity of helminths to alter the course of infections by viruses, protozoa, bacteria and fungi [5, 13, 14, 23, 46]; however, the influence of *A. suum* infection on malaria needs to be elucidated [4, 15, 34]. In this context, this study was designed and carried out as a pioneering study to evaluate the impact of initial *A. suum* infection on probable concomitant malaria infection. Thus, we used an MA-ARDS experimental model with *P. berghei* [31, 37, 38] and an *Ascaris* larvae model [26, 36, 40].

This study showed that *P. berghei* and *A. suum* concomitant infection causes a serious risk to the host. The results showed that coinfection induced higher morbidity and earlier mortality than monoinfections. Manifestations of severe malarial morbidity are a consequence of some pathogenic processes, such as erythrocyte destruction, the toxin-mediated activation of cytokine cascades, and infected cell sequestration in blood capillaries. In humans, severe morbidity occurs in children < 5 years of age, and the fetuses of infected pregnant women experience the most morbidity and mortality from the disease [28, 47, 48]. However, it is important to note that *A. suum* monoinfection also exhibits morbidity in infected mice, as evidenced by the loss of body weight, which is recovered after larvae migration through the intestine, which is a common event in this model of infection [26]. Morbidity associated with larvae migration is also present in pig and human (definitive hosts) infections and defines the acute phase of ascariasis [21, 40, 49–54]. The similarity between the pattern of body weight loss between coinfected- and *A. suum-*monoinfected animals indicates that *Ascaris* influences the morbidity of coinfected animals.

The results obtained on the *Ascaris* lung parasite load show that coinfected animals presented alterations in pulmonary larvae migration that were associated with a decrease in larvae recovered in the lung parenchyma and an increase in larvae recovered in the airways, suggesting that there was a change in the migration of larvae through the lungs in these animals. Despite this finding, there were no changes in *Plasmodium* parasitaemia in the coinfection group relative to that in the monoinfected group, suggesting that the immune modulation is driven by *Plasmodium*.

Given the changes in the larvae migration of *Ascaris* in the lungs, the next step was to understand the pulmonary pathology of coinfected animals. Larvae migration is a process that generates mechanical damage to the lung tissue, resulting in the formation of haemorrhagic areas and oedema. In addition, during this process, the production of secreted/excreted larvae antigens [54–56] in the tissue promotes local inflammation that involves strong leukocyte recruitment, especially of eosinophils, as a response to effectively control the larvae in the tissue [25, 30]. In this larvae ascariasis experimental model, it was possible to verify these haemorrhagic and inflammatory phenomena. However, during coinfection, the low levels of leukocyte recruitment to the lung tissue indicates that this response was impaired, as shown by the low levels of NAG, MPO and EPO activities in the organ. The increase in larvae migration in coinfected animals generated an increase in haemorrhagic phenomena, mainly in the airways of these animals, where a larger number of larvae was recovered.

To better understand the immunological response involved in the *Plasmodium-Ascaris* interaction, the cellular immune response in the lungs of these animals was evaluated. The results suggested that the immune response was dampened in coinfected mice. The overall immune response elicited during coinfection seems to be driven by *Plasmodium* infection due to the similarity to the *Plasmodium* monoinfection profile, except for IL-6 and IL-33 production, which were significantly higher in coinfected animals. The increased production of IL-6 and IL-33 might be explained by the influence of the host’s response to *Ascaris* infection. IL-6 is an important cytokine secreted by inflammatory cells and epithelial cells of the lungs against allergens and viral, bacterial and parasitic infections. This mediator is considered an important regulator of effector CD4^+^ T cells, promoting IL-4 production during Th2 differentiation, inhibiting Th1 differentiation and, together with TGF-β, promoting Th17 cell differentiation [57, 58]. As seen in preliminary studies [26, 36, 45], during primary exposure to *Ascaris*, the larvae in the lungs elicit strong innate and adaptive local responses characterized by increased levels of IL-4, IL-5, IL-6 and IL-33 cytokines. The increase in IL-33 production in coinfected animals reinforces the importance of this mediator in the activation of type 2 innate lymphoid cells (ILC 2) for establishing the helminth response [59, 60]. Based on this finding, the increase in IL-6 and IL-33 in the lungs of coinfected animals suggests that there was a response to the increased larvae migration in the organ as a consequence of *Plasmodium*-driven modulation.

In contrast, when assessing the circulating cell profile in the blood of coinfected animals, a significant increase in the total number of circulating leukocytes, characterized by lymphocyte, monocyte and neutrophil populations, was observed. Although the response is not as robust in primary infection as reinfection [36], it was possible to verify that there was a significant increase in eosinophils in the bloodstream in *Ascaris*-monoinfected mice; eosinophils are an important cell type in the protective response against *Ascaris* and other helminths [26, 36, 60, 61]. Previous studies [62] have shown the importance of eosinophilia in the control of *Plasmodium* parasitaemia; however, the response produced during the acute phase of primary infection by *Ascaris* was ineffective in containing the protozoan in this study. The systemic and local responses induced by *Plasmodium* caused low responsiveness to the larvae in the lung, which culminated in a sepsis-like process, leading to the early death of the animals [63]. In addition, the significant reductions in the total erythrocyte count and haemoglobin and platelet levels in coinfection are directly related to the dynamics of malaria infection, including the constant rupture of red blood cells during the erythrocytic parasite cycle and the destruction of infected and uninfected red blood cells, platelets, and other blood components in other organs, such as the spleen and liver [64–68]. Given these findings, the low immune responsiveness in the lung tissue coupled with low airway leukocyte recruitment in contrast to the increased cellularity in the circulation might be explained by the exhaustion/anergy phenomena induced by *Plasmodium* spp. as an escape mechanism to stay in the host [69].

During the past decade, many studies have examined the impact of malaria coinfection with helminths and other microorganisms, but the results have been contradictory. In a model of simultaneous coinfection by *A. suum* and *Vaccinia* virus (VACV) [15], viral replication was favored, resulting in a decrease in the number of larvae recovered from the lung. This coinfection caused an increase in pulmonary inflammation combined with the absence of circulating CD8^+^ and CD4^+^ T cells producing IFN-γ, potentiating the pathology associated with the virus by negatively modulating the specific immune response of VACV, which resulted in an increased mortality rate of coinfected animals. Another context was described by Wang et al*.* [56], who investigated different preceding inocula of *Schistosoma japonicum* cercariae associated with high and low densities of the *P. berghei* ANKA strain. This study showed that coinfection with a larger *S. japonicum* inoculum and lower *P. berghei* density provided an increase in parasitaemia with higher production of IL-4, IL-5, IL-13, TGF-β and Tregs, decreased levels of IFN-γ, a lower percentage of CD4^+^ and CD8^+^ T cells in the spleen and the infiltration of CD8^+^ T cells in the brain. This response profile resulted in an improved survival rate of these animals compared to that in animals coinfected with less *S. japonicum* inoculum. However, in this latest model of coinfection, there were no significant changes in cytokine levels. Another study of concomitant infection was reported by Helmby [70], in which it was demonstrated that C57BL/6J mice infected with *Heligmosomoides polygyrus* and *Plasmodium chabaudi* presented higher mortality and increased *Plasmodium* parasitaemia. The authors also investigated the importance of the time of infection and found that there were no significant differences in coinfection with previous exposure to *H. polygyrus* compared to simultaneous coinfection. In this model, coinfection resulted in very pronounced liver damage compared to helminth monoinfection, where the imbalance of the immune response was evident due to the high expression of the IFN-γ, IL-17 and IL-22 cytokines in the liver tissue. Moriyasu et al*.* [71] demonstrated in their model of coinfection with *Plasmodium yoelii* and previous infection with *Schistosoma mansoni* that coinfected animals showed a reduction in *Plasmodium* parasitaemia in the liver, but this did not alter the mortality rate, independent of the mouse strain (BALB/c, C57BL/6J or CBA). Therefore, the outcome of coinfections with malaria and helminths depends on the species of parasites involved, the parasitic burden on the host, the stage of helminthic infection (acute versus chronic) and the host's exposure history for each parasite [34, 56, 72–74].

Based on these findings, the low inflammation in the pulmonary parenchyma and the low levels of leukocyte recruitment in the airways enhanced the haemorrhage and oedema caused by the increase in larvae migration during coinfection and are determinants of changes in the pulmonary physiology of these animals. The implications of *Ascaris* larvae migration in the lungs in pulmonary physiology was described in previous studies [36], and this migration results in the loss of pulmonary elasticity as a result of increased septum thickness due to leukocyte infiltration, oedema and haemorrhage in the parenchyma and airways. However, after during coinfection and are determinants of changes in the pulmonary physiology of these animals migration, *Ascaris*-infected mice tended to recover, in contrast to coinfected animals (Fig. 1C). Coinfection causes major respiratory impairment due to the *Plasmodium*-driven immune response, which causes an imbalance of the local response against the larvae, favoring faster larvae migration through the lung parenchyma. The increased migration of *Ascaris* led to an increase in haemorrhage in the lung parenchyma and airways. Haemorrhage is the main phenomenon leading to airflow obstruction in the lungs of coinfected animals, as suggested by the significantly lower compliance, increased resistance and decreased Tiffeneau index (FEV50/FVC), even in *Ascaris*-monoinfected animals, suggesting that there was greater pulmonary impairment in coinfection.

Due to study bias, since only concomitant coinfection was studied, it was not possible to understand what happens in other endemic scenarios, such as early exposure to *Ascaris* before *Plasmodium* or vice versa. Furthermore, due to the limitations of the model, it was not possible to understand what happens in coinfection during the chronic phase of ascariasis and to identify the specific mechanism of death of the animals in the present study.

## Conclusions

In summary, the results of this study suggest that *Plasmodium-Ascaris* coinfection is harmful to the host. Coinfection may potentiate *Ascaris*-associated lung pathology by dampening the *Ascaris*-specific immune response, resulting in the early death of these animals. Moreover, this study provides evidence on how helminth and protozoan coinfection may influence the course of monoinfection, enhancing the public health impact for geographically overlapping endemic areas for both pathogens. In this context, it is necessary to better understand the immunological mechanisms involved in coinfection, with the aim of understanding the role of immune cells in pulmonary pathology during coinfection with these parasites.

## Supplementary Information

**Additional file 1: Table S1.** Histopathological scoring system for mouse lungs.

## Data Availability

The datasets generated and/or analysed during the current study are available in the Figshare repository, https://doi.org/10.6084/m9.figshare.11714400.

## Abbreviations

BAL: Bronchoalveolar lavage
dpi: Day postinfection
ELISA: Enzyme-linked immunosorbent assay
EPO: Eosinophil peroxidase
IFN-γ: Interferon gamma
IL: Interleukin
iRBCs: Infected red blood cells
MA-ARDS: Malaria-associated acute respiratory distress syndrome
MPO: Neutrophil myeloperoxidase
NAG: *N*-acetylglucosaminidase
PBS: Phosphate-buffered saline
TGF-β: Transforming growth factor beta
TNF: Tumour necrosis factor
