# Supplementary material for: Concomitant experimental coinfection by Plasmodium berghei NK65-NY and Ascaris suum downregulates the Ascaris-specific immune response and potentiates Ascaris-associated lung pathology
Source: Malar J. 2021 Jul 1;20:296. doi: 10.1186/s12936-021-03824-w (PMC8248286; doi:10.1186/s12936-021-03824-w)
Supplement: Supplementary file 1 — Additional file 1: Table S1. Histopathological scoring system for mouse lungs. [file 12936_2021_3824_MOESM1_ESM.docx]

**S1 Table** **Histopathological scoring system for mouse lungs**

| Score 1  Airways Inflammation Score /6  0 = Lack of inflammatory cells around airways - Absent  1 = Some airways have small numbers of cells - Mild  2 = Some airways have significant inflammation - Moderate  3 = Majority of airways have some inflammation - Marked  4 = Majority of airways are significantly inflamed - Severe  5 = All of airways are completely inflamed - Whole  Score 2  Vascular Inflammation Score /6  0 = Lack of inflammatory cells around vessels - Absent  1 = Some vessels have small numbers of cells - Mild  2 = Some vessels have significant inflammation - Moderate  3 = Majority of vessels have some inflammation - Marked  4 = Majority of vessels are significantly inflamed - Severe  5 = All of vessels are completely inflamed - Whole  Score 3  Parenchymal Inflammation (at 10X magnification) Score /6  0 = <1% affected  1 = 1-9% affected  2 = 10-29% affected  3 = 30-49% affected  4 = 50-69% affected  5 = >70% affected  Score 4  Hemorrhage score Score /4  0 = No hemorrhage  1 = Small hemorrhage zones - slight  2 = Presence of significant hemorrhagic areas - moderate  3 = Presence of exuberant hemorrhagic zones - intense) |
| --- |
